# Curvature and self-assembly of semi-conducting nanoplatelets

**DOI:** 10.1038/s42004-021-00621-z

**Published:** 2022-01-12

**Authors:** Lilian Guillemeney, Laurent Lermusiaux, Guillaume Landaburu, Benoit Wagnon, Benjamin Abécassis

**Affiliations:** Univ. Lyon, ENS de Lyon, CNRS, Laboratoire de Chimie, 69342 Lyon, France

**Keywords:** Two-dimensional materials, Quantum dots, Optical materials, Self-assembly

## Abstract

Semi-conducting nanoplatelets are two-dimensional nanoparticles whose thickness is in the nanometer range and controlled at the atomic level. They have come up as a new category of nanomaterial with promising optical properties due to the efficient confinement of the exciton in the thickness direction. In this perspective, we first describe the various conformations of these 2D nanoparticles which display a variety of bent and curved geometries and present experimental evidences linking their curvature to the ligand-induced surface stress. We then focus on the assembly of nanoplatelets into superlattices to harness the particularly efficient energy transfer between them, and discuss different approaches that allow for directional control and positioning in large scale assemblies. We emphasize on the fundamental aspects of the assembly at the colloidal scale in which ligand-induced forces and kinetic effects play a dominant role. Finally, we highlight the collective properties that can be studied when a fine control over the assembly of nanoplatelets is achieved.

## Abstract (web summary)

Two-dimensional colloidal nanoplatelets can assemble into materials with promising optical properties, and the influence of local curvature on these properties is an area of active interest. Here, the relationship between nanoplatelet geometry, self-assembly, and collective properties is reviewed.

## Introduction

**Fig. 1:**
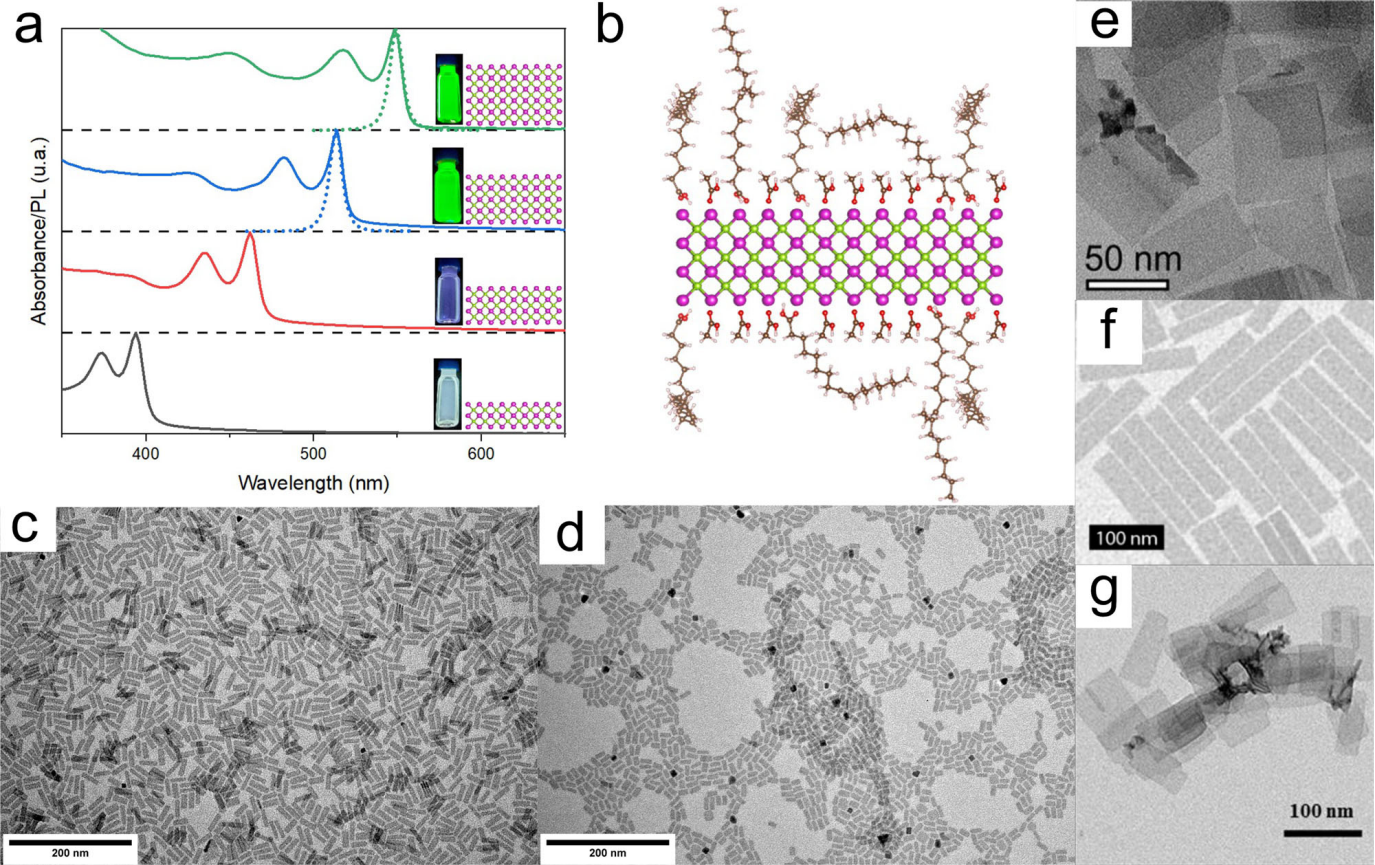
Optical Properties, surface ligands, and electron microscopic images of flat semiconducting NPLs. **a** UV-vis absorbance (full line) and fluorescence (dotted line) spectra of 2 to 5 ML CdSe NPLs (from bottom to top). **b** Schematic representation of the cross-section of a 3 ML CdSe NPL with oleic acid and acetate ligands on its surface. Magenta corresponds to Cd, green to Se, red to O, brown to C, and white to H. **c**–**g** TEM images of **c** 4 ML CdSe NPLs, **d** 5 ML CdSe NPLs, and **e** 4 ML CdSe NPLs with a monolayer of CdS. Reprinted with permission from ref. ^29^. Copyright 2013 American Chemical Society. **f** 1.2 nm-thick PbS nanosheets. Reprinted with permission from ref. ^44^. Copyright 2019 American Chemical Society. **g** 2.2 nm-thick CdTe NPLs. Reprinted with permission from ref. ^18^. Copyright 2013 American Chemical Society.

Colloidal nanoplatelets (NPLs) are two-dimensional (2D) nanocrystals covered on both sides by a layer of surfactant bound to their surface by a functional group^1–3^. The ligands provide stability to the crystalline structure, made of a single or a few atomic layers, which would not be thermodynamically stable otherwise. They are usually synthesized from organo-metallic precursors in solution and therefore differ from other types of 2D materials obtained from exfoliation of a layered bulk sample, such as graphene. In this review, we focus on ultrathin NPLs with a thickness in the range of the nanometer. Strikingly, their thickness can be controlled at the atomic level. In this regard, this property is similar to those of magic-sized clusters that have preferred atomic compositions with deep energy minima. Therefore, NPLs possess a structure that is perfectly defined in one dimension (the thickness), however, the two other geometrical dimensions display some polydispersity, as commonly observed for other types of colloidal nanocrystals. Semiconducting NPLs have been the topic of intense research due to their outstanding optical properties. Their perfectly defined thickness coupled to the small refractive index of the ligand monolayer (ML) results in strong quantum confinement in one direction and a density of states of a quantum well. Their emission and absorption linewidths are thus extremely small (Fig. 1a). Furthermore, they display giant oscillator strength at cryogenic temperatures^4–6^, very low lasing thresholds^7–9^, and very fast Förster resonance energy transfer (FRET) rates^10,11^, which could be exploited in a wide range of optoelectronic applications.

Several previous reviews have been dedicated to this type of nanoparticle. The review of Nasilowski et al. spans a large variety of materials going from semiconductors to metals and transition metal dichalcogenides while recalling the growth thermodynamics, the different anisotropic growth mechanisms, and potential applications^12^. Dating from around the same time, two conspectus articles focused preferentially on semiconducting metal chalcogenides NPLs^13,14^. The concept of ultrathin nanomaterial, either in one-dimensional (1D) or 2D, was well introduced and described by the de Mello group in a perspective^15^. More recently, a review on colloidal quantum wells detailed their use in optoelectronic devices^16^.

Here we focus on semiconducting core-only NPLs with an emphasis on II–VI semiconductors such as CdX (X = Se, Te, or S) in the zinc blende or wurtzite crystallographic phase. Among these materials, CdSe is by far the most studied and serves as a model system. We first briefly recall useful facts regarding their synthesis, structure, and surface chemistry. We then focus on the curvature and conformation of 2D NPLs as they actually are not always flat. For instance, they can adopt different shapes depending on their lateral dimensions, surface chemistry, and crystallographic orientation. Beyond thermal fluctuations that cause out-of-plane deformations of atomic MLs, as observed in graphene^17^, curvature control of 2D nanomaterials is an important topic since optical and electronic properties are modified by structural deformations that break the symmetry of the particle. For colloidal NPLs, the current understanding of the origin of these conformations points toward the critical role of the stress induced by surface ligands in relation to the highly deformable character of the NPLs due to their extreme slenderness. We also outline how this can impact their optical properties. Afterward, we review several approaches developed to achieve assembly of semiconducting NPLs into larger structures with controlled orientation. Assembly of nanoparticles can be directed using molecular templates such as polymers, guided by a fine-tuning of the colloidal forces in solution or provoked using external forces, such as interfacial forces. Moreover, self-assembly can be performed either in solution, possibly followed by subsequent deposition of the large structures on another substrate, or directly during particle deposition onto a substrate. We distinguish NPLs assembled side by side with their planes parallel to the substrate or stacked face to face with their edges in contact with the substrate. Finally, we outline the current challenges in the field and point towards future directions.

## Model system: CdSe NPLs

Zinc blende CdX (X = Se, S, Te) NPLs are synthesized from cadmium carboxylates and several different selenium precursors (selenium mesh, Se-ODE or trioctylphosphine-Se)^3,18,19^. Their top and bottom layers are [001] polar facets and they are usually designated as *N* MLs for a Cd_*N*+1_X_*N*_ composition along their thickness direction (Fig. 1). Some authors prefer using the *N* + 0.5 convention to identify the number of MLs. Hence, when referring to 3 ML NPLs, other sources employ 3.5 ML to identify the same type of particle. A common feature in most of the syntheses is the presence of cadmium acetate alongside other longer ligands. Recent studies have shown that even though it is used in most of the current protocols, it is not mandatory and NPLs can be obtained without acetate^20,21^. Amine ligands were also thought to favor the formation of wurtzite CdSe NPLs but a recent study showed that 3 ML zinc blende curved NPLs could be obtained in diamine solvents^22^. The thickness of the NPL increases with the synthesis temperature with 2 and 3 ML thin NPL appearing between 100 and 220 °C^23–25^ while temperatures in the 240–280 °C range are necessary to synthesize thicker NPLs with 5–7 MLs. The use of chlorine precursors has also proven efficient to produce thick NPLs^26,27^.

Among the different geometric characteristics of NPLs (thickness and two lateral dimensions), only the thickness can be controlled at the atomic level. Dispersions of NPLs with a given thickness are routinely obtained after synthesis and size-selective purification but fine independent control over the two lateral dimensions is still in its infancy. A given synthetic protocol often affords limited flexibility on the lateral dimensions. The next few examples show various approaches used to control particle dimensions. First, it was shown that the water content of the cadmium precursor has an impact on the aspect ratio of 4 ML NPLs with dry cadmium acetate yielding slender ribbon-like shapes, whereas the addition of water decreased the aspect ratio to square-like shapes with similar lateral dimensions, in the 10–15 nm range^28^. Second, slow and gradual injection of precursors leads to extended 3 ML sheets with large lateral dimensions (>50 nm)^29,30^, whereas hot injections syntheses—during which all the precursors are present at the beginning of the synthesis and short-chain carboxylic acids or carboxylates are injected—yield ribbon-like geometries with one lateral dimension larger than the other^21,25^. For 3 ML NPLs, changing the carbon chain length of the Cd precursor is an efficient way to tune the lateral dimension of the NPL^25^.

The surface chemistry of NPLs is an important feature that has a strong influence on their curvature and self-assembly (Fig. 1b). Recent reports have studied this aspect for zinc blende CdSe NPLs using advanced nuclear magnetic resonance (NMR) and infrared spectroscopy techniques and showed that the Cd-rich surfaces are coated with carboxylate groups^31–34^ through a Cd(O_2_CR)_2_ binding motif. The measured carboxylate density is close to the maximum density of functional groups allowed by the amount of cadmium at the surface (5.4 groups/nm^2^). Interestingly, all the ligands used in the synthesis are found to be present at the surface including acetate groups^33^. In the case of wurtzite CdSe NPLs, the Burho group has shown in detail that various ligand exchanges can be performed to replace the native L-type amine ligands by other functional groups such as metal carboxylates^35^ or protic acids^36^. For both crystallographic structures, metal halides were found to bind the surface efficiently^30,37,38^.

## Curvature and conformation

**Fig. 2:**
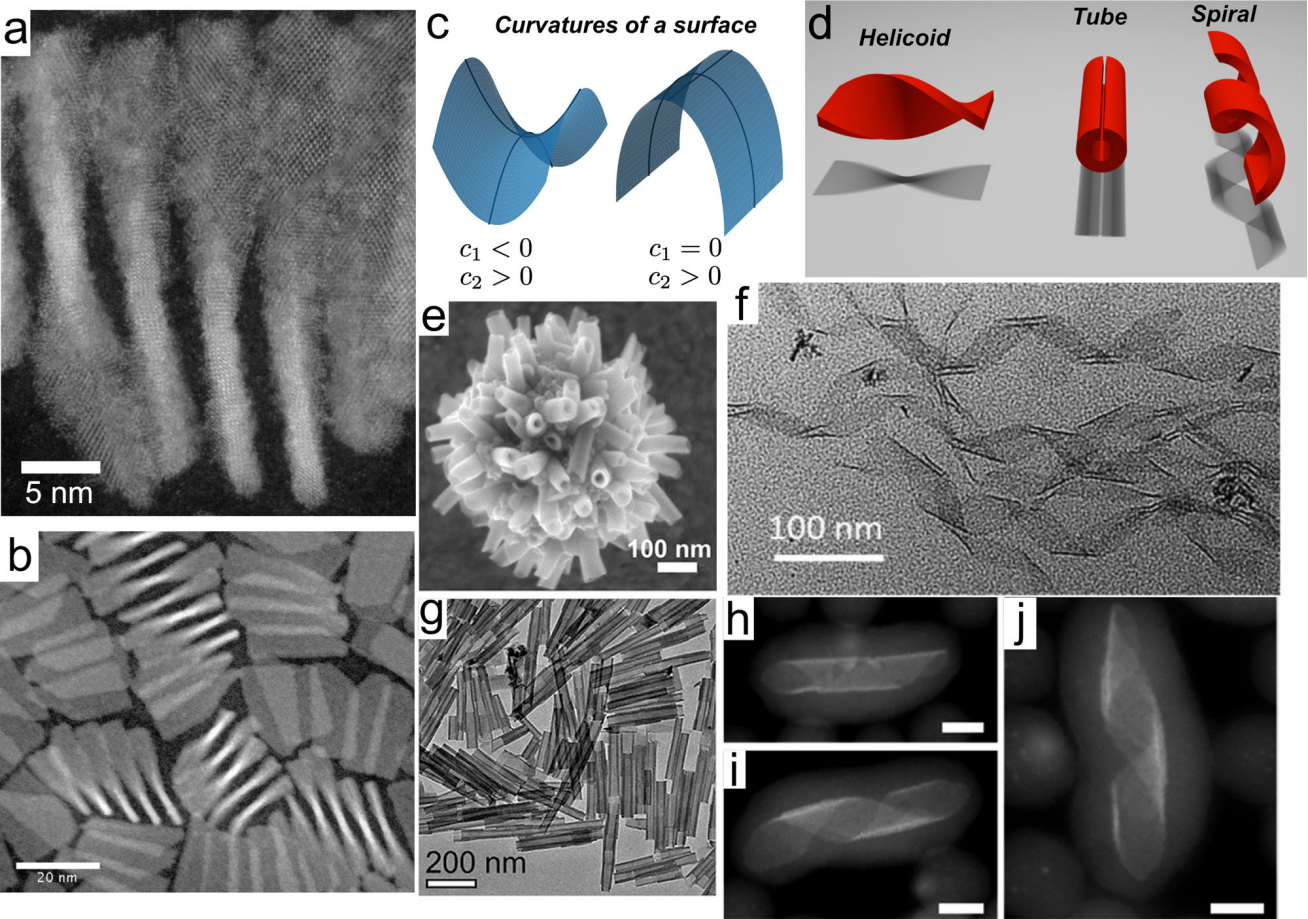
Geometry of curved CdSe NPLs. **a**, **b** STEM images of 5 ML helicoidal NPL^46^. **c** Representation of the principal local curvatures of a surface. Left: the two principal curvatures have opposite signs leading to a negative Gaussian curvature *κ*_2_ = *c*_1_ × *c*_2_. Right: the Gaussian curvature is zero. **d** Different conformations of a ribbon. The shadows below the different shapes correspond to their projections on a surface and should thus resemble the TEM images of the corresponding particles. **e**, **g** CdSe 3 ML NPLs folded into tubular structures. Reprinted with permission from refs. ^22,29^. Copyright 2019 and 2013 American Chemical Society. **f** 3 ML NPLs adopting a spiral ribbon geometry. Reprinted with permission from ref. ^49^. Copyright 2019 American Chemical Society. **h**–**j** Spiral ribbons coated with a silica shell. Reprinted with permission from ref. ^47^. Copyright 2014 American Chemical Society. Scale bars correspond to 5 nm.

An important geometrical characteristic of 2D NPLs is their curvature as it describes the way NPLs bend and curl and *in fine*, dictates their 3D shape. Knowing and controlling their spatial configuration is crucial for several aspects such as their optical properties and their self-assembly. Experimentally, their shape is determined by electron microscopy in which we observe the particle’s projection on a surface. We note that, however, if small out-of-plane deformations can be detected using advanced electron microscopy^17^, routine characterization techniques are not suitable to detect small curvatures with respect to the lateral dimensions of the NPL. In order to describe, understand and compare the shapes of these nanostructures, we can use the rigorous conceptual framework which has been developed to describe the geometry of soft matter interfaces^39,40^. The concept of principal curvatures is useful to classify the different geometries that a flexible plane can adopt. At any point, a surface bares a curvature along two orthogonal directions, called principal directions. The product of these two principal curvatures is the Gaussian curvature, while their average is the mean curvature (Fig. 2c). Canonical shapes can be classified depending on the value of their Gaussian and mean curvatures (Fig. 2d). For example, a tube or a spiral ribbon has a finite mean curvature but a zero Gaussian curvature since one of their principal curvature is zero. In contrast, an helicoïd has a straight centerline, a zero mean curvature, and a non-zero Gaussian curvature. Such an object is mathematically defined as a minimal surface, i.e., one that minimizes its area while being subjected to a deformation. On such a surface, principal curvatures are equal in modulus but opposite in sign in every point, leading to a mean curvature equal to zero.

A high diversity of NPL shapes can be found in the literature although most semiconducting NPLs are flat (Fig. 1c–g). This is the case for CdTe NPLs even with lateral dimensions reaching hundreds of nanometers (Fig. 1g)^18^, hexagonal InSe^41^, In_2_S_3_^42^, In_2_Se_3_^43^, PbTe, and rectangular^44^ (Fig. 1f) and square^45^ PbS nanosheets. CdSe NPLs with thicknesses >4 MLs are usually flat (Fig. 1c–e), but in some instances, they display an original helicoidal shape with a non-zero Gaussian curvature^46^. Thin 2 or 3 CdSe NPLs are usually curved^29,47–49^. Depending on their lateral dimensions, they can appear as folded sheets^24,29^ (Fig. 2a, d) or helices (Fig. 2f, h–j)^30,47,49^. In all cases, they do not display any Gaussian curvature and one of their principal curvatures is zero. Furthermore, they show a preferred bending axis and always display the same folding direction along the [110] axis of the zinc blende structure. Their final geometry depends on the orientation of their edges with respect to this crystallographic axis and their lateral dimensions. Interestingly, the curvature does not appear after the synthesis but the NPLs grow curved as recently proven by time-resolved small-angle X-ray scattering (SAXS)^48^. This mechanism can result in the “closing” of the curved sheets into nanotubes (Fig. 2e, g)^22^. Interestingly, wurtzite CdSe NPLs of similar thickness do not adopt curved geometries^2^. Cylindrical curvatures are also observed in In_2_S_3_ coiled nanoribbons^50^, which close into In_2_S_3_ nanotubes when the reaction temperature is increased^51^. Finally, crumpled sheets have been observed in the case of In_2_S_3_ NPLs synthesized at high temperature^52,53^.

### Geometry, elasticity, and ligand-induced surface stress

This structural diversity requests a physical explanation. We propose that the surfactant monolayer at the surface of the NPL induces surface stress, which deforms its inorganic core. It has been known for a long time that adsorbates induce surface stress on crystalline surfaces^54,55^. When organic ligands are adsorbed onto a surface, organometallic bonds are created, which deforms the surface of the crystal. The same kind of phenomenon occurs during epitaxy, in the region where two crystals with different lattice parameters are bound together, the atomic arrangement at the interface accommodates this difference through strain. The lattice parameter of the denser crystal increases while the other one decreases. The magnitude of the surface strain at the interface depends on the original mismatch between the two crystals. The ligand-induced surface stress was also reported for thiol self-assembled MLs on gold cantilevers^56^. In Berger et al., the alkanethiol ligands used were shown to self-assemble into a well-ordered densely packed film at the surface of the nanoparticle, each alkyl chain exhibiting a tilted orientation compared to the normal of the surface in order to optimize inter-chains interactions. This ligand ML imposes compressive surface stress whose strength, which increases linearly with the length of the alkyl chain and with the number of adsorbed molecules, can reach hundreds of mM/N. In semiconducting nanocrystals, tuning the surface stress by the accurate choice of the surface ligand has been realized in the case of quantum dots^57^. By choosing a *σ*-donor or a *π*-acceptor ligand, it was shown that the surface reconstruction of quantum dots could be achieved accurately and that different ligands could apply compressive or tensile stress^57^.

Ligand-induced variation of surface stress and its effect on the shape and optical properties of NPLs has been reported in several publications in the last few years. Controlling and shifting the shape of a given NPL can be achieved by tuning the NPL surface chemistry. Bouet et al. have shown that growing a ML of CdS can unfold tube-like CdSe NPLs to yield flat NPLs (Fig. 1e)^29^. In several other studies, the chemical function of the surface ligand was changed. For example, Antanovitch et al. exchanged native carboyxlate ligands with hexadecanethiol and hexadecylphosphonic acid on the surface of CdSe NPLs^58^. They observed a spectral shift in the absorbance spectra directly linked to the structural change of CdSe NPL induced by the change in surface stress. Ligand exchange induces a tetragonal deformation of the NPL, i.e. a shrinkage in their lateral direction and an expansion in their thickness direction. Thus, surface ligands directly affect the crystalline structure of the NPLs and red-shift their excitonic band gap, up to 240 meV. Another example has recently been described for CdTe nanosheets where the ligand exchange of native oleic acid by thiol leads to their folding into tubes^59^. Thiol attachment on the basal plane of the nanosheets leads to the formation of an external CdS ML. The lattice parameters of this CdS layer largely mismatch those of the CdTe (by about 11.4%), resulting in a tensile strain in the CdS layer and a compression strain in the CdTe core layer. These strains result in a spontaneous relaxation of the nanosheets by rolling on themselves. The folding direction of the nanosheets may also depend on the orientation of thiol molecules on the surface and on the intrinsic elastic properties of zinc blende CdTe structure. The folding of CdTe nanosheets induces a redshift of the excitonic transitions due to a thickness increase (two additional sulfur planes) and the onset of strain under thiol attachment. Inversely, a ligand exchange of native-carboxylate ligands by halide ligands reduces the surface stress imposed on 3 ML CdSe NPLs and leads to their unfolding^30^. Indeed, carboxylate-capped NPLs initially fold into helices as carboxylate ligands impose tensile stress on the top and bottom facets of the NPLs. When halide ligands are exchanged, the stress decreases, and the NPL unfolds, increasing significantly the quantum yield of CdSe zinc blende NPL by up to 70%, because of better surface passivation of the NPL surface.

Shape-shifting can also occur without changing the functional group covering the NPL surface. For example, rectangular 5 ML CdSe NPL, which are linked to carboxylate ligands after their synthesis and purification, change their shape upon the addition of oleic acid. From a flat configuration, they shape-shift to yield helicoids when a certain quantity of free oleic acid is added in solution^46^. This shows that ligand-induced stress does not only depend on its anchoring via the functional group but that ligand-ligand interactions, *via* their aliphatic chain at the surface, can also play a role. This was evidenced by changes in the FTIR signal and reflects that ordering of the alkyl chain layer likely impacts surface stress.

In the case of wurtzite NPL, the amine ligands originally present at the surface undergo fast and facile exchange with neutral metal halides. Though the shape of the NPL does not seem to be affected, this ligand exchange induces a large shift in the extinction features^37,38^.

Overall, these examples highlight the fact that a given NPL inorganic core can adopt different conformations depending on the organic surface ligand coating their surface. Due to their very small thickness, the energy needed to bend and twist a NPL is typically of the order of 10 eV. This order of magnitude is estimated by calculating the energy needed to twist a 1 nm-thick NPL^46^. We can also evaluate the surface stress needed to bend a 1 nm-thick NPL, given by $\frac{E{t}^{2}}{6R(1-\nu )},$ where *E* is the bending modulus of the material, *ν* the Poisson ratio, *t* the thickness of the NPL, and *R* the curvature. Using the mechanical properties of bulk CdSe and a radius of curvature of 50 nm yields typical surface stress of 250 mN/m. This is the same order of magnitude as surface stresses measured on a gold cantilever imposed by a thiol ML^56^. These estimates are also consistent with a recent report showing that Ag nanoplates are significantly deformed by weak van der Waal forces^60^.

## Chiral NPLs

Chiral optical properties have also been observed in wurtzite and zinc blende CdSe NPLs when their surfaces are functionalized by cysteine ligands^24,38,61,62^. The intensity of the optical activity of CdSe NPL coated with L- or D-cysteine depends on the thickness, lateral dimensions, and core-crown character of the NPL^61^. The very high *g*-factors, reaching 5.3 × 10^−3^ for wurtzite 3 ML NPLs^62^ and 3 × 10^−3^ for zinc blende 2 ML NPLs, are among the largest measured in quantum dots systems^63^. In their very thorough and insightful article, Gao et al. show that zinc blende and wurtzite CdSe NPLs have distinct circular dichroism signatures and attribute this to a different coupling between cysteine and the NPL surface^62^. Their theoretical approach is based on the two-group model which explains the optical activity by the hybridization of the molecular orbitals of the chiral ligand with those of the NPLs considered here as simple chromophores. This approach contrasts with other theoretical reports that consider the chiroptical properties of chiral NPLs^64–66^. In those, several shapes are considered such as nanosprings or tapered nanoscrolls, and the absorption and circular dichroism are then calculated for interband and intraband transitions using a full quantum-mechanical approach of the band structure.

### Towards a rational design of shape-changing chiral NPLs

A wealth of evidence has shown that ligands can induce (chiral) shape changes to ultrathin crystalline NPLs. However, there is a long road toward the rational design of 3D shapes via controlled folding and stretching of nanosheets. One of the fundamental knowledge gaps separating us from this long-term achievement is that the link between the molecular structure of the ligand and the stress that a ML applies to the top and bottom surfaces of the NPL is not yet understood. It likely depends on the organometallic bonding between the particle’s surface atoms and the functional group of the ligand but also on the detailed organization of the ligands self-assembled as a ML. A related problem is to understand how the surface stress imposed by the ligands transduces into strain within the crystalline core of the NPL and how it affects the NPL shape. Solving this strain-stress mechanical problem should benefit from the work of a large scientific community working on the elasticity and mechanics of thin sheets^67^, with specificities pertaining to the nanoscale. An open question is to determine whether the theoretical framework of elasticity^68^ is still valid at the nanoscale. The spatial configuration of NPL must result from an interplay between their mechanical energy and the preferred geometrical curvature dictated by the surface stress. As such, ligand-coated NPLs are frustrated systems that must adapt to a set of incompatible constraints^69^. Another open question concerns more specifically chirality. Circular dichroïsm emerges from the coupling between the chiral electronic state of the ligands and the band structure of the semiconductor but it is not clear yet what roles play the shape of the inorganic core with respect to electronic coupling with chiral ligands. More research is thus needed in this direction to disentangle the two effects.

## NPL assemblies

### Colloidal interactions between NPLs

NPL self-assemblies can be controlled by both kinetic and thermodynamic effects. Therefore, a fundamental comprehension of the colloidal interaction between NPLs is primordial but current theories remain incomplete. At the nanoscale, the conceptual framework developed in the past century for micron-scale colloids does not hold due to the non-additivity of the forces at this scale^70^. In the classical Derjaguin–Landau–Verwey–Overbeek view of colloidal interactions, only van der Waals attraction between surfaces and electrostatic interactions are taken into account^71^. At the nanoscale, and specifically in the case of NPLs, three other types of interaction are likely to be relevant. First, dipolar interactions can play an important role^72^. Permanent ground state dipoles result from an imbalance of charges within nanocrystals and, even though their physical origin is still debated^73^, recent experimental measurements have shown that NPLs can bear dipolar moments as high as 245 D^74^. Depletion interactions have been shown to be relevant for nanorods^75^ and should also be taken into account for NPL. This attraction of entropic origin appears when free molecules or particles are present in solution alongside the particles of interest^76^. The magnitude of depletion attraction is directly proportional to the concentration of depletant in the solution. Finally, ligand-mediated van der Waals forces are likely to be of great importance. Computer simulations^77,78^ and experiments have shown that the structure of the self-assembled ML at the surface of nanoparticles is key in their inter-particle interaction potential and colloidal stability^79^. More specifically, recent reports have explored experimentally and via molecular dynamics simulations the effect of alkane solvents, ligand chain length, and inorganic core radius on the interparticle mean force potential^80,81^. For small cores, ligand/ligand interactions dominate and both their magnitude and repulsive or attractive nature are closely related to the structure of the self-assembled ML. Ordered MLs, which are generally obtained for long alkyl chains and low temperatures, induce a strong attraction while disordered ligands favor dispersed states. This rationale explains why branched “entropic” ligands or mixed self-assembled ML (with the ligand of various alkyl lengths) provide nanocrystals with high dispersibility up to 100 mg/mL^82,83^. Although these studies have been performed for spherical and rod-like particles, they are relevant for NPL as for a given volume of nanoparticle the area of ligand/ligand interparticle contact is maximum for 2D geometries^84,85^. Ligand-induced interactions are likely to depend finely on the structure of the self-assembled ML. Beyond the chain length, changes in the density of ligands (which depends on synthesis and purification procedure) can impact the self-assembly. Insaturation also influences the order/disorder transitions in the self-assembled ML with saturated ligands being more prone to ordering^86^. Finally, unbound ligands can impact the assembly of particles via two different mechanisms: it can swell the ligand shell through van der Waals interactions with the chains of the bound ligand^87^ but can also induce a depletion attraction between nanocrystals. The latter is likely to occur at higher concentrations than the former since depletion attractions are proportional to the volume fraction of free object in solution, whereas swelling of the ligand shell requires a very small amount of molecules, typically of the same order of magnitude as the concentration of bound ligand, i.e., in the mM range.

### Self-assembly at liquid surfaces

**Fig. 3:**
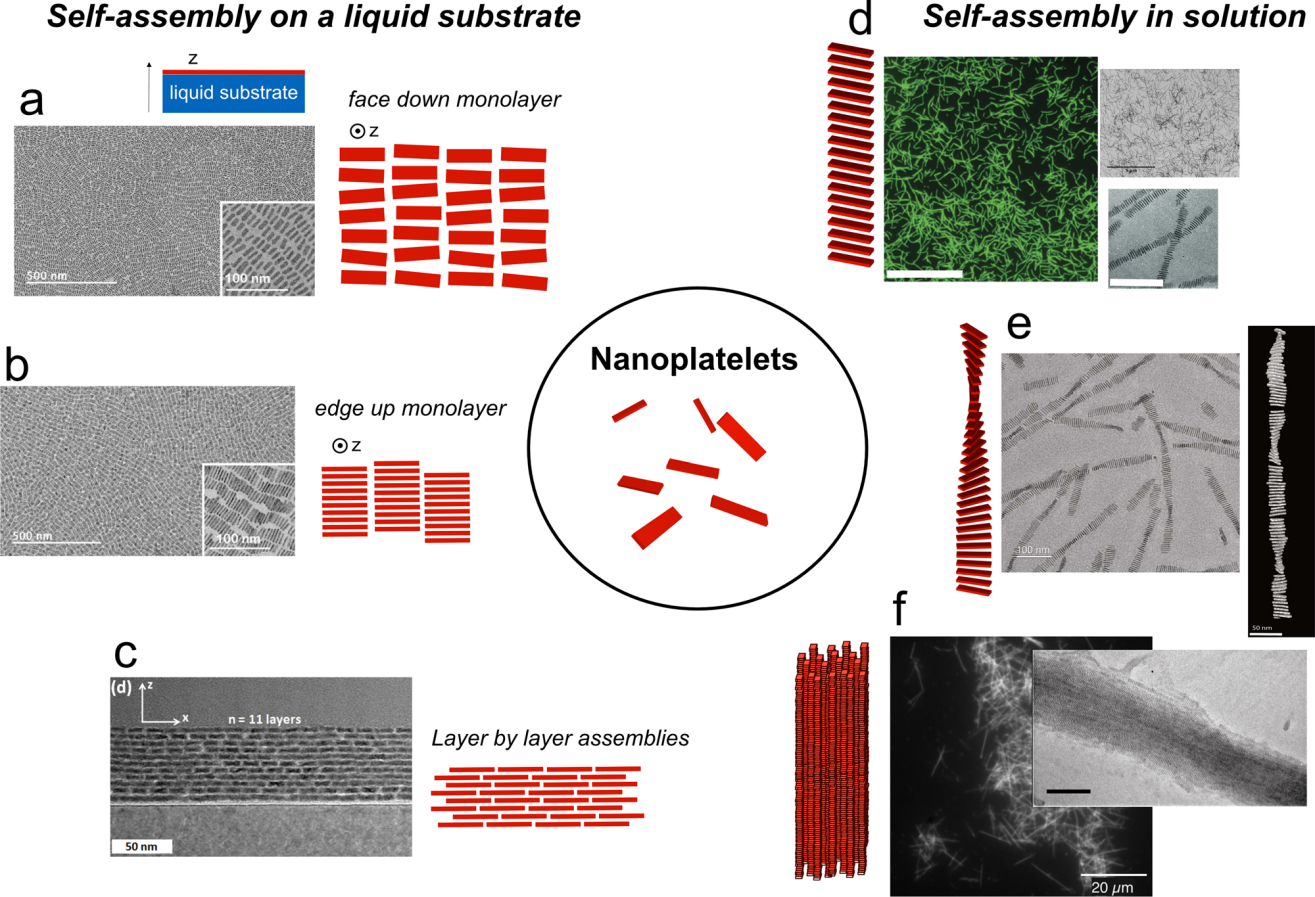
Self-assembled structures of CdSe NPLs. Starting for colloidal NPLs (central scheme), self-assemblies can be performed on a liquid substrate, for example, by deposition on non-miscible solvent such as ethylene glycol (**a**–**c**), or in solution (**d**–**f**). Single monolayers with **a** face-down or **b** edge-up orientations can be obtained by tuning the self-assembly conditions. **c** Multilayers can also be obtained by repeating the deposition process several times. Adapted with permission from refs. ^89,92^. Copyright 2017 and 2020 American Chemical Society. **d**, **e** Polymer-like structures can be obtained through evaporation of colloidal dispersions in the presence of oleic acid. Scale bars correspond to 5 μm for the confocal image (left) and 20 nm for the bottom right TEM image. Reprinted with permissions from refs. ^46,95^. Copyright 2016 John Wiley and Sons. **f** Larger structures result from the fast destabilization by an antisolvent. Scale bar in the TEM image (right) corresponds to 100 nm. Adapted with permission from ref. ^94^. Copyright 2014 American Chemical Society.

An experimental approach pioneered by Murray et al.^88^ has been used to assemble NPLs into dense MLs with different kinds of ordering (Fig. 3a–c). A dispersion of NPLs in a volatile solvent is spread onto another solvent in which the NPLs are not soluble. This sub-phase is often composed of ethylene glycol which has a low vapor pressure. Then, the solvent in which the NPLs are dispersed evaporates and the interface serves as a template for the assembly. Films formed by this technique can later be transferred to a different substrate for further structural or optical characterizations. This strategy has been efficiently used in the last years for the self-assembly of a large variety of nanoparticles and mixtures to achieve unusual particles organization including quasi-crystalline assemblies. In the case of NPLs, 4 ML and 5 ML CdSe NPLs have been assembled into MLs with domain sizes comprised of hundreds of nanometers and tens of micrometers^89^. Depending on the content of the sub-phase and on the evaporation conditions, edge-up or face-down configurations can be achieved (Fig. 3a, b). In the first case, the NPL plane is perpendicular to the liquid-liquid interface while in the second configuration, the NPLs are in mutual contact through their edges. Interestingly, tuning the oleic acid concentration in the sub-phase has an important effect on the assembly with edge-up assembly for 0.42 mM of oleic acid and face-down configuration for larger (4.2 mM) oleic acid concentrations. Recently, Momper et al.^90^ explored how evaporation kinetics could also tune the self-assembled structures of 4 ML NPL. Using an acetonitrile sub-phase and alkanes of different chain lengths as the solvent, they showed that the edge-up configuration is the thermodynamically stable configuration while face-down assembly is the result of kinetic trapping for fast evaporation rates. They also discovered that the adventitious presence of octadecene solvent and oleic acid that had not been cleaned during the purification of the NPLs had dramatic effects on the self-assembly with predominantly edge-up configurations when these impurities were present in the mM and μM regimes, respectively. A similar evaporating scheme was used to study FRET between NPLs and quantum dots^91^. Recently, this assembly method has been extended towards layer-by-layer assembly to produce organized films^92^.

### Self-assembly in solution

Assembly can also occur in solution through tuning of interactions between NPLs (Fig. 3d–f). After synthesis and without dedicated self-assembly strategies, NPLs have a strong tendency to stack in a co-facial fashion with their ligand MLs in close contact. This stacking also occurs spontaneously in solution after purification or ligand exchange as evidenced by peaks in the SAXS pattern typical of lamellar assemblies^18,45,93^. The distance between the stacked NPLs can thus be tuned by modifying the ligand chain length. In the case of wurtzite CdSe NPLs, this stacking behavior is attributed to the synthesis mechanism during which an amine surfactant mesophase serves as a template for magic-sized clusters to assemble. It results in a bundling of the NPLs, which extends on the micron scale^2^.

The colloidal stability of NPL dispersions depends on several key parameters such as the amount of free ligand in solution, the length of the ligand carbon chain, or the presence of trace amounts of water in the solvent^93^. These results give new parameters toward a more rigorous understanding of the inter-NPL forces, but additional studies are necessary in this area. Controlled destabilization of NPL dispersions can lead to new super-structures within which the NPLs are organized. Our group has previously focused on developing such strategies (Fig. 3d–f). For example, we have observed that upon addition of small quantities of anti-solvent, such as ethanol, square 4 ML CdSe NPLs could form long anisotropic supra-crystals whose length could reach several micrometers^94^. In these needles, the NPLs stack in a 1D fashion one on top of each other and exhibit a crystalline order. In the lateral direction, the needles are composed of 10 to 20 NPLs while along their long direction, they extend over hundreds of NPL (Fig. 3f). More recently, we showed that the slow evaporation of colloidal dispersion in the presence of excess oleic acid leads to ribbons of NPLs also stacked face to face but laterally composed of only one NPL (Fig. 3d)^95^. This high aspect ratio provides the assembly with some flexibility and fragility which renders them amenable to further growth by the addition of supplementary monomers. The length of the assemblies can be finely tuned by controlling the amount of oleic acid added at the beginning of the drying process. Twisted ribbons in which the NPLs rotate along the thread axis can also be formed by following successive addition of oleic acid during the evaporation of a 5 ML colloidal suspension of NPLs (Fig. 3e)^46^. The size of the threads can also reach several micrometers in length and depend directly on the amount of oleic acid added. This original chiral geometry results from the frustration between the stacking of the NPL and their tendency to bend under the mechanical constraints of the ligands as described in the section “Towards a rational design of shape-changing chiral NPLs.” Stress release propagates along the thread and results in a clear pitch that recalls cholesteric liquid crystals. Similar structures were obtained with 4 ML NPLs, also after the addition of oleic acid and drying. In this case, the pitch was shown to depend on the lateral dimension of the NPLs^96^.

### Polymer–NPL composites

Another strategy to orient NPLs in space is to use a polymer matrix. The NPLs are first mixed with the polymer in a solvent and a composite film is formed as a result of the evaporation of the solvent^97–99^. The use of styrene-butadiene-styrene block copolymers and poly-(butyl-methacrylate) co-lisobutyllmethacrilate leads to films with the appropriate mechanical properties so that, upon uniaxial stretching of the composite film, the NPLs align parallel to the stretching direction following the hydrophobic blocks of the copolymer. SAXS measurements showed that an order parameter of 0.6 can be attained for a 100% elongation of the composite film. This figure is close to order parameters typically achieved in liquid crystals. Interestingly, the emission properties of the film are also anisotropic when strain is applied. Another valuable feature of this approach is its reversibility since the film retains its original shape and dimensions when the strain is released.

### Photonic properties of NPL assemblies

One of the main interests in controlling the self-assembly of NPLs, and particularly their relative orientation, is to harness their outstanding optical properties. Due to their 2D shape, NPLs absorb and emit light differently depending on its polarization. Oriented self-assembled NPLs can be used to probe these anisotropic optical properties. For example, the anisotropic needles described in the section “Self-assembly in solution” display highly polarized photoluminescence consistent with an emission dipole oriented within the plane of the NPL^94^. Ma et al. have oriented individual NPLs by using an etched glass surface to tune its surface roughness^100^. When deposited on this rough substrate, NPLs are all the more tilted that the surface is rough and even if the orientation of individual NPL is not known, variation of the mean angle between the NPL plane and the excitation source is achieved. By these means, it was shown that light absorption in CdSe/CdS core–shell NPLs is isotropic but that emission is strongly polarized. Using back focal plane imaging on MLs of oriented CdSe NPLs, researchers have shown that emission dipoles are highly oriented in the plane of the NPL^89,101^, but that they are degenerate in the two directions within the plane. Recent Fourier imaging experiments on self-assembled CdSe NPL chains also confirmed a small anisotropy within the plane with a slightly larger dipolar component in the direction of the larger dimension. A small out-of-plane dipolar component has also been measured in this study^102^.

**Fig. 4:**
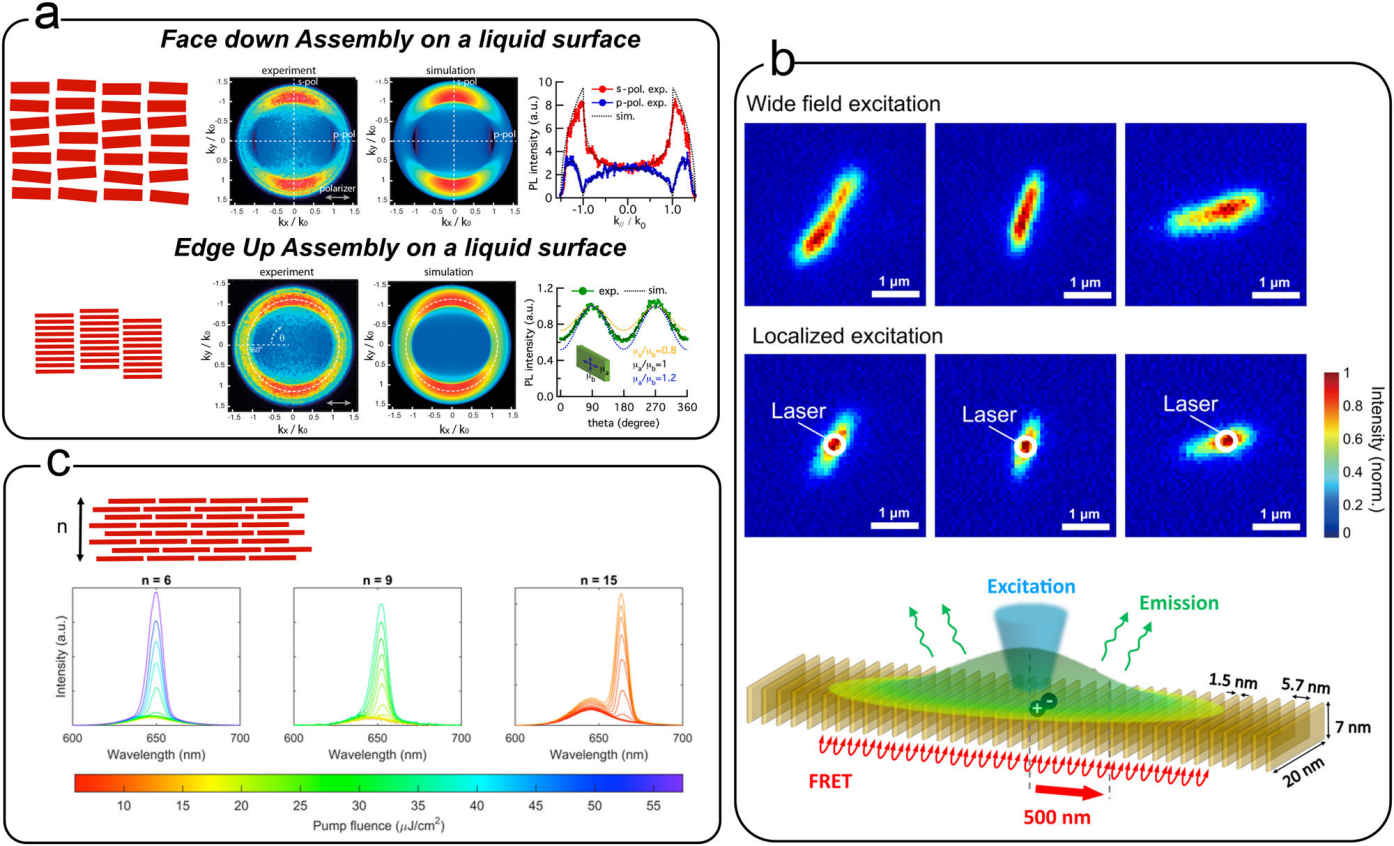
Optical properties of assembled NPLs. **a** Anisotropic emission properties of CdSe NPLs assembled at a liquid interface. Back focal plane imaging (experiment and theory) for two different assembly configurations (face down and edge up) showing the orientation of the emission dipole. Adapted from ref. ^89^. Copyright American Chemical Society 2017. **b** (bottom) Scheme of the FRET occurring in face-to-face stacked nanoplatelets. (middle–top) Microfluorescence images showing assemblies of stacked 5 ML NPLs. (top) Wide-field excitation shows the elongated shape of the assemblies. (middle) Localized laser excitation shows that the fluorescence extends beyond the excitation area (white circle) and demonstrates that the energy transfer occurs on large length scales. Adapted with permission from ref. ^113^. Copyright American Chemical Society 2020. **c** Effect of the number of layers on the emission spectra of NPL films. The onset of a second emission feature as the pump fluence intensity is increased is typical of amplified spontaneous emission. The gain threshold decreases as the number of layers increases. Adapted with permission from ref. ^91^. Copyright 2019 American Chemical Society.

The photoluminescence dynamics and the lasing properties of the NPLs are also strongly impacted when they are assembled. For example, the lasing threshold decreases as the number of layers increases in stacked assemblies^92^ (Fig. 4c) due to increased field confinement of the optical mode within the NPL slab whose optical index is larger than that of air and the substrate. Stacking decreases the photoluminescence lifetime and the quantum yield compared to isolated NPLs, due to a fast and efficient FRET between NPLs^11^. This was proven by comparing, for suspended or assembled NPLs, the photoluminescence spectra and lifetime measurements in single and multiple excitonic regimes. It was also observed that an energy transfer is possible between NPLs of the same thickness (homo-FRET) and that the transfer is faster than hetero-FRET between NPLs of different thicknesses. Since homo-FRET between neighboring NPLs occurs on the picosecond timescale, it happens prior to radiative recombination and even outpaces Auger recombination, one of the preferred non-radiative processes in semiconducting nanoparticles^103^. Within NPL stacks, these energy transfers can occur between NPL before radiative or non-radiative recombination happens. Non-radiative events take place at a defective “dark” NPL within the stack which acts as an exciton sink for the whole assembly yielding to an overall decrease in the quantum yield^10,104,105^. Several numerical models account for this energy transfer, sometimes with predictions in very good agreement with the experimental data. For example, a model based on a Markov chain produced a very good estimate of quantum yields and lifetimes of assembled NPLs^106^. Assembly is also suspected to be at the origin of the low energy peak visible in the photoluminescence of NPLs at low temperature^107^. However, this explanation is called into question by recent experiments which attribute this peak to the trion emission at low temperature^108–110^. Other explanations have also been put forward such a the formation of an excimer^111^ or a p-state emission^112^. Beyond ensemble measurements in solution, energy transfer between NPLs within isolated stacks was recently evidenced using microfluorescence experiments (see Fig. 4b). When a NPL chain is lit with a laser spot, fluorescence is detected well beyond the location of the laser spot, proving unambiguously that excitons travel along the chain before recombining and generating a photon. In this work, the experimental results were well described by a simple model and excitation migrations on distances over 500 nm were measured, corresponding to FRET hopping over >90 NPLs^113^.

### Surface chemistry, shape control, and assembly processes toward controlled superstructures

Although researchers have made progress toward the rational design of NPL assemblies, important challenges still remain. One pitfall comes from the non-perfect reproducibility of 4 and 5 ML CdSe NPL syntheses. Current protocols are robust, in the sense that they always yield the desired product, but the purity, reaction yield, and NPL shape vary from batch to batch. This is caused by several factors. First, the synthesis is highly dependent on the cadmium carboxylate precursor. Its preparation and storage modalities impact the outcome of the synthesis in terms of purity and shape control. The amount of adventitious water present is a plausible explanation since it is known to control the lateral dimension of the NPL^28,114^. Second, both the precise moment at which the Cd acetate is injected during the heating ramp and the heating rate have an influence on the quality of the final product. Finally, some molecules in the solution have been identified as impurities which later impact the self-assembly. This is the case of oleic acid, which is injected in large amounts at the end of the synthesis, as well as unpurified amounts of octadecene (the solvent of the synthesis)^90^. Both molecules can act as depletants to induce face-to-face stacking or as swelling agents of the ligand shell, modifying the inter-ligand interaction potential. Depletion forces can also be induced by polymerized species, which can result for example from the polymerization of octadecene at high temperature^115^. However, the heating duration and maximum temperature used in typical NPL syntheses are typically low enough to limit this phenomenon. We also note that the geometrical shape of the NPL is important in the self-assembly process. Edge-to-edge assembly in solution, and at interfaces, is facilitated by sharp edges and square shapes^116^.

Future progress will come from better control of the synthesis of NPLs but also from the fundamental understanding of the colloidal forces at play during the assembly process. To do so, the community needs to investigate further the structure of the self-assembled ML that coats the NPLs to get an atomic view of the NPL-solvent interface depending on the synthesis and purification protocols. Recent efforts toward this goal have emerged but our insight on the link between the structure of the self-assembled ML and self-assembly is still sketchy. Getting such an atomic view will necessitate the mobilization of a vast array of high-resolution techniques such as small-angle neutron scattering, NMR, or second-harmonic generation coupled to rigorous synthesis and purification protocols. Since the self-assembly of nanoparticles is often controlled by kinetics, it is also critical to control not only the purity and the geometry of the building blocks but also to tame the self-assembly process that can include injections and drying steps^90^. The fundamental aspects underlying, for example, the dynamics of the triple line during the evaporation of colloidal dispersion at a liquid/liquid/gas interface are important to understand in order to get predictive and reproducible assembly protocols.

## Outlook

Our journey through the synthesis, curvature control, and self-assembly of colloidal NPLs taught us that these systems have an ambivalent character and behave in many aspects more like supramolecular systems than immutable solid-state crystals. As the scale decreases toward a few atoms, concepts relevant for molecular assemblies start to be pertinent. This is beautifully illustrated by the recent discovery that small magic-sized CdS clusters can switch between two distinct isomers under modification of their surface ligand motifs^117^. There are also more and more experimental data showing that colloidal interactions between NPLs are dominated by weak supramolecular forces, producing fragile structures that can break under the effect of thermal agitation. NPLs can change their shape under very small surface stress induced by organic ligand and still display outstanding (chiral) optical properties that are unique to heavy elements and their strong interaction with light. Future progress will come from a better fundamental understanding of the parameters controlling their synthesis, twisting, and assembly. This requires new insights from a diverse set of areas ranging from mechanics at the nanoscale to surface chemistry of semiconductors and soft matter.
